# *Macacine alphaherpesvirus* 1 (B Virus) Infection in Humans, Japan, 2019

**DOI:** 10.3201/eid3001.230435

**Published:** 2024-01

**Authors:** Souichi Yamada, Harutaka Katano, Yuko Sato, Tadaki Suzuki, Akihiko Uda, Keita Ishijima, Motoi Suzuki, Daigo Yamada, Shizuko Harada, Hitomi Kinoshita, Phu Hoang Anh Nguyen, Hideki Ebihara, Ken Maeda, Masayuki Saijo, Shuetsu Fukushi

**Affiliations:** National Institute of Infectious Diseases, Tokyo, Japan (S. Yamada, H. Katano, Y. Sato, T. Suzuki, A. Uda, K. Ishijima, M. Suzuki, S. Harada, H. Kinoshita, P.H.A. Nguyen, H. Ebihara, K. Maeda, M. Saijo, S. Fukushi);; Ministry of Health, Labor and Welfare, Tokyo (D. Yamada);; Health and Welfare Bureau, Sapporo, Japan (M. Saijo)

**Keywords:** *Macacine alphaherpesvirus* 1, B virus, viruses, zoonoses, Japan

## Abstract

Two human patients with *Macacine alphaherpesvirus* 1 infection were identified in Japan in 2019. Both patients had worked at the same company, which had a macaque facility. The rhesus-genotype B virus genome was detected in cerebrospinal fluid samples from both patients.

The herpesvirus *Macacine alphaherpesvirus* 1 (herpes B virus, or B virus) is ubiquitous in macaque monkeys. Although macaque monkeys do not usually show symptoms when infected with B virus, humans show severe disease, including encephalitis and encephalomyelitis, and death frequently results from infection with B virus from monkeys (*1*). However, B virus infection of humans is rare. Infection can occur after being bitten or scratched by a macaque monkey that is actively shedding the virus or through direct contact with bodily fluids or contaminated laboratory materials. Since B virus infection was first reported in 1934, more than 50 cases have been reported, mainly in North America, and 29 cases have been confirmed, including a recent case in China (*2*,*3*). In most cases in which a specific macaque species was identified, patients had been exposed to rhesus macaques, rather than other species of monkey (e.g., cynomolgus macaques, African green monkeys, Vervet monkeys, or Sykes monkeys) (*2*).

In this study, 2 patients in Japan with chronic and long-term neurologic diseases were tested for B virus infection; the B virus genome was detected in cerebrospinal fluid (CSF). Both patients had worked at a macaque facility in Japan. To maintain confidentiality and privacy, we report no personal information, or information about the clinical course or the working environment. All protocols and procedures were approved by the research ethics committee of the National Institute of Infectious Diseases for the use of human subjects (approval no. 1314). We confirmed B virus infection in both patients by using molecular assay and, in one patient, by also using immunohistochemical analysis. We describe molecular and immunohistochemical findings in the 2 patients. 

## The Study

Patient 1 worked in the macaque facility at a pharmaceutical research company. In 2019, the patient was hospitalized for headache, fever, and deterioration of consciousness. CSF samples were collected at the time of hospitalization and sent to the National Institute of Infectious Diseases (Tokyo, Japan), where we tested them for B virus infection. We extracted total DNA from the CSF samples and tested the samples by using real-time PCR with primers and a fluorescent probe targeting the B virus gB gene: forward primer, 5′- CGTGGCCAGGTAGTACTGCAC-3′; reverse primer, 5′- CTCGTTCCGCTTCTCCTCGTC-3′); AND fluorescent-labeled probe, 5′- FAM-TAGCGCCGGAGGAA-MGB-3′. The reaction mixture (total volume 25 µL) contained 12.5 µL TaqMan Universal PCR Master Mix (ThermoFisher, https://www.thermofisher.com), 2.0 µg/mL of sonicated salmon sperm DNA, 0.2 µmol/L of each primer and fluorescein amidite–labeled probe, and 4.0 µL of extracted DNA. We subjected the reaction mixture to real-time PCR by using an ABI-7500 Fast Real-Time PCR System (ThermoFisher). The reaction conditions were as follows: 50°C for 2 min and 95°C for 10 min, followed by 40 cycles of 95°C for 15 s and 60°C for 60 s. We performed real-time PCR targeting the B virus gG gene as described previously (*4*). We performed conventional PCR by using primers targeting a gB gene region conserved among primate herpes viruses (herpes-PCR) as described previously (*5*). We detected the gB and gG genes in the CSF samples at a concentration of 5.1 × 10^5^ copies/mL for gB and 7.6 × 10^5^ copies/mL for gG. Sequencing of the 364-bp herpes-PCR product revealed 100% identity with a B virus sequence from GenBank (accession no. LC637778).

**Figure 1:**
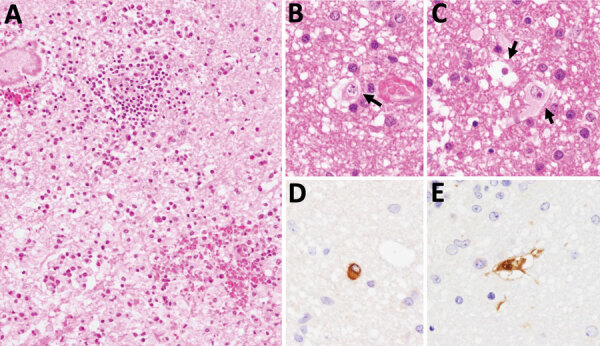
Brain biopsy from patient (patient 2) with *Macacine alphaherpesvirus* 1 (herpes B virus) infection, Japan, 2019. A–C) Inflammatory cell infiltration and hemorrhage observed around blood vessels in the cerebellar white matter. Arrows indicate nuclear inclusion bodies (B, C). Hematoxylin and eosin stain. D, E) Immunohistochemical analysis using B virus gB mouse monoclonal (clone 19B6) (D) and an B virus rabbit polyclonal (E) antibodies as the primary antibodies. Original magnification × 200 for all images.

Patient 2 had been diagnosed with chronic neurologic disease. The patient was tested for B virus infection because they worked at the same facility as patient 1 and had been working with macaques. In 2014, patient 2 was admitted to hospital with fever, headache, and neurologic symptoms. Brain biopsy was performed, but no pathogen was detected at that time. In 2019, after identification of patient 1, we collected a CSF sample and tested it for B virus infection in addition to testing a paraffin-embedded section of the brain biopsy collected in 2014. Real-time PCR of the CSF detected the gB (3.5 × 10^5^ copies/mL) and gG genes (2.0 × 10^6^ copies/mL) of B virus. Nucleotide sequencing of the 364-bp herpes-PCR product confirmed that it had 100% identity with the sequence detected in patient 1. Furthermore, real-time PCR of the DNA extracted from the paraffin-embedded section of brain tissue biopsied in 2014 was positive for the B virus gG gene. Real-time PCR did not detect herpes simplex virus 1, herpes simplex virus 2, or varicella zoster virus. Histologic analysis of the brain biopsy revealed inflammatory cell infiltration and hemorrhage in the white matter of the cerebellum (Figure 1). Careful observation revealed the presence of inclusion bodies in the nuclei. Immunohistochemical analysis using B virus rabbit polyclonal and gB mouse monoclonal antibodies generated positive signals in cells with inclusion bodies (*6*).

## Conclusions

**Figure 2:**
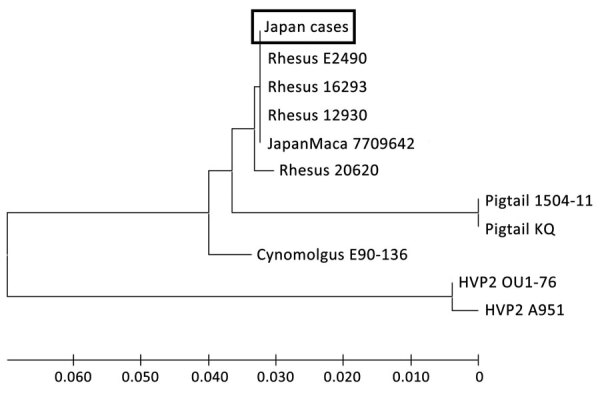
Phylogenetic tree of *Macacine alphaherpesvirus* 1 (herpes B virus) gB gene in 2 patients with B virus infection, Japan, 2019. Nucleotide sequences of the herpes-specific PCR products (364 bp) from the 2 patients were aligned with the corresponding region of the B virus gB gene from GenBank (accession no. LC637778 for virus from patient 1 and LC637779 for virus from patient 2). Phylogenetic tree with HVP2 as an outgroup constructed using the neighbor-joining method. Scale bar indicates number of nucleotide substitutions per site. HVP2, herpesvirus papio 2.

From our results, we concluded that patients 1 and 2 had been infected with B virus. Although the 2 patients worked in the facility managing imported macaques, there was no direct evidence that they were infected from imported macaques. Given that no epidemiologic link between the 2 patients had been recorded, it seems that they were infected independently. Nucleotide sequence analysis identified B virus genotypes known to be carried by the macaque species (*7*,*8*). Recently, a case of human B virus infection was identified in China; however, the genotype was not identified (*3*). Phylogenetic analysis of the gB gene indicated that B virus from the 2 patients in Japan clustered with the genotype in rhesus macaques (Figure 2). The full-length nucleotide sequences of the gG, gB, and gJ genes also were obtained from the CSF of patient 2 and also were classified by phylogenetic analysis as the rhesus B virus genotype (data not shown). Although there is no direct epidemiologic evidence of zoonotic transmission of rhesus B virus in the monkey facility, the results indicated that workers in such facilities are at risk for infection with rhesus B virus infection, which is pathogenic in humans.
